# Chemotherapy exacerbates ovarian cancer cell migration and cancer stem cell-like characteristics through GLI1

**DOI:** 10.1038/s41416-020-0825-7

**Published:** 2020-04-03

**Authors:** Yawei Zhao, Meihui He, Lianzhi Cui, Mohan Gao, Min Zhang, Fengli Yue, Tongfei Shi, Xuehan Yang, Yue Pan, Xiao Zheng, Yong Jia, Dan Shao, Jing Li, Kan He, Li Chen

**Affiliations:** 1 Department of Pharmacology, Nanomedicine Engineering Laboratory of Jilin Province, College of Basic Medical Sciences, Jilin University, Changchun, 130021 China; 2 Clinical Laboratory, Jilin Cancer Hospital, Changchun, 130012 China; 3 School of Nursing, Jilin University, Changchun, 130021 China; 4 Department of Biomedical Engineering, Columbia University, New York, NY 10027 USA

**Keywords:** Ovarian cancer, Cancer metabolism

## Abstract

**Background:**

Despite the great clinical response to the first-line chemotherapeutics, metastasis still happens among most of the ovarian cancer patients within 2 years.

**Methods:**

Using multiple human ovarian cancer cell lines, a transwell co-culture system of the carboplatin or VP-16-challenged feeder and receptor cells was established to demonstrate the chemotherapy-exacerbated migration. The migration and cancer stem cell (CSC)-like characteristics were determined by wound healing, transwell migration, flow cytometry and sphere formation. mRNA and protein expression were identified by qPCR and western blot. Bioinformatics analysis was used to investigate the differentially expressed genes. GLI1 expression in tissue samples was analysed by immunohistochemistry.

**Results:**

Chemotherapy was found to not only kill tumour cells, but also trigger the induction of CSC-like traits and the migration of ovarian cancer cells. EMT markers Vimentin and Snail in receptor cells were upregulated in the microenvironment of chemotherapy-challenged feeder cells. The transcription factor GLI1 was upregulated by chemotherapy in both clinical samples and cell lines. Follow-up functional experiments illustrated that inhibiting GLI1 reversed the chemotherapy-exacerbated CSC-like traits, including CD44 and CD133, as well as prevented the migration of ovarian cancer cells.

**Conclusions:**

Targeting GLI1 may improve clinical benefits in the chemotherapy-exacerbated metastasis in ovarian cancer treatment.

## Background

Ovarian cancer is the leading cause of death among gynaecological cancers.^1^ Platinum, paclitaxel or the combination of platinum and paclitaxel-based chemotherapy are used as the first-line chemotherapeutics for the clinical management of ovarian cancer.^2^ Despite the achievement of a complete clinical response, recurrence and metastasis are still observed among ~70% of the patients within 2 years after initial diagnosis, which is responsible for the high mortality of ovarian cancer.^3^ Although chemotherapy is one of the major efficient medical interventions, our and other recent studies have found that it may also induce intratumoural or systemic changes, which may paradoxically exacerbate the proliferation and dissemination of cancer cells.^4^ Different chemotherapy drugs, including paclitaxel and carboplatin, were observed to induce the migration of cancer cells in different kinds of cancers.^5–7^ The secretion of cytokine and inflammatory mediators, such as the C–X–C chemokine receptor type-4 (CXCR4) and prostaglandinE2 (PGE2) that are essential for inducing the epithelial–mesenchymal transition (EMT) of cancer, was found to be elevated in the tumour microenvironment after chemotherapy treatments, indicating that the altered tumour microenvironment by chemotherapy may contribute to the chemotherapy-induced metastasis.^8,9^

Cancer stem cells (CSCs) are reported to exacerbate the initiation, differentiation, maintenance, dissemination, drug resistance and recurrence of tumour in various types of cancers.^10^ Some recognised markers, such as CD133, ALDH, CD44 and CD117, are used to identify ovarian cancer stem cells (OCSCs).^11^ The upregulation of CSC markers has been reported to induce the process of EMT and the migration of tumour cells in different cancers.^12,13^ Recently, an in vivo study has demonstrated that the short-term treatment of cisplatin and paclitaxel resulted in an increase in CSC-like traits and led to an increased tumour burden and metastatic potential, suggesting that the first-line chemotherapy treatment may result in the increase of CSC-like phenotype in ovarian tumours, and trigger the facilitation of the metastasis and relapse of ovarian cancer.^14^ However, the early molecular mechanisms triggered by the chemotherapy treatments that increased the cancer stem cell-like characteristics and ultimately induced the migration ability of ovarian cancer cells are still not well established.

The GLI family are transcription factors of the Hedgehog (Hh) signalling, including GLI1, GLI2 and GLI3. GLI1 usually acts as a strong activator of targets, whereas GLI2 and 3 have dual functions as repressor or activator depending on different circumstances.^15^ Hh signalling is essential for embryonic development and maintaining many tissues and organs.^16^ Studies have also observed an activation of Hh signalling in pancreatic, glioma and gastric cancers, functioning in maintaining the characteristics of cancer stem cells.^16–18^ GLI1 is known to be associated with the CSC-like phenotype formation in gastric cancer,^16^ and its inhibitor GANT61 can inhibit the growth of pancreatic cancer stem cells.^18^ Other studies have linked GLI1 with the EMT and invasion of tumour cells.^19^

In our study, the Transwell co-culture system of the chemotherapy drug-treated and -untreated ovarian cancer cells was established, to investigate the indirect influence of chemotherapy on the CSC-like characteristics of ovarian cancer cells by altering the tumour microenvironment. The migration and sphere formation ability were significantly enhanced in the microenvironment of the chemotherapy-challenged dying cells in two different ovarian cancer cell lines. The EMT markers as well as the pluripotency-associated gene BMI1, which was regulated by transcription factor GLI1, were upregulated by chemotherapy treatments. GLI1 was clinically associated with chemotherapy treatments and the survival of ovarian cancer patients. Downregulation of GLI1 reversed the chemotherapy-induced CSC-like characteristics and migration of ovarian cancer cells. Our study emphasised the paradox that in spite of the crucial role chemotherapy played in the medical treatments of ovarian cancer, it may also trigger the increase in the CSC-like characteristics of tumour cells and induce the metastasis of ovarian cancer. A thorough understanding of the molecular mechanisms of the chemotherapy-exacerbated migration of ovarian cancer cells may contribute to the development of new strategies of better chemotherapy treatments.

## Methods

### Cell culture

The human ovarian cancer cell lines SKOV3, A2780 and KURAMOCHI were cultured in RPMI 1640 (Cat#11875119, Gibco, USA) supplemented with 10% foetal bovine serum (FBS, Cat#10099141, Gibco, USA) and 1% penicillin–streptomycin liquid (Cat#P1400, Solarbio Life Sciences, China). Cells were incubated at 37 °C in 5% CO_2_–95% air atmosphere.

### Treatment of ovarian cancer cells with chemotherapy drugs and Transwell co-culture system

Treatment of ovarian cancer cells with chemotherapy drugs and the Transwell co-culture system was consistent with our previous studies with minor modification.^4,20^ The human ovarian cancer cell lines SKOV-3 and A2780 were seeded into 6- (10^5^ cells per well) or 24-well (2.5 × 10^4^ cells per well) plates and incubated for 24 h and allowed to reach 60–65% confluence. The cells were then treated with a single dose of 100 μM carboplatin (Cat#S1215, Selleck Chemicals, USA) or 5 μM VP-16 (Etoposide Injection, Qilu pharmaceutical Co., LTD) for 24 h (Day 0). Then the cells were rinsed with phosphate-buffered saline (PBS) twice, and fresh culture medium was added into each well. For SKOV-3 or A2780 cells treated with carboplatin, the cells were cultured for another 3 days (Day1–Day 3); for SKOV-3 treated with VP-16, the cells were cultured for another 6 days (Day1–Day 6); for A2780 treated with VP-16, the cells were cultured for another 5 days (Day 1–Day 5). The majority of cells died gradually during the culture period after the chemotherapy drugs were removed from the system, and the cells and conditioned medium of the culture system were used for future experiments.

The 4.0-μm pore-size semipermeable membrane filter chambers (Cat#3450, Corning, USA) were used in the Transwell co-culture system. First, the SKOV-3 cells or A2780 cells (as feeder cells) were treated by the chemotherapy drugs consistent with the conditions mentioned above for 24 h. In the meantime, SKOV-3 cells or A2780 cells (4 × 10^4^ cells per well, as receptor cells) were seeded into six-well plates and allowed to adhere overnight. Then the same number of feeder cells in each group in 2 mL of fresh culture medium were seeded into the upper inserts that were placed into the six-well plates seeded with receptor cells. The cells in the upper and lower chambers were co-cultured for 3–5 days consistent with the conditions mentioned above. Then the receptor cells in the lower chamber were collected for western blot or quantitative real-time PCR analysis. In the RNA interference experiments using siRNA of GLI1, the chambers with chemotherapy-treated or -untreated feeder cells were inserted into the wells with siRNA or negative control (NC)-transfected receptor cells. After 48 h of co-culture, the receptor cells in the lower chamber were collected for quantitative real-time PCR analysis. After 72 h of co-culture, the receptor cells in the lower chamber were collected for western blot analysis.

### Cell migration assay

Cell migration assay was carried out as previously described.^21^ Briefly, the Transwell chambers (Cat#3464, Corning, USA) with 8.0-μm pore-size semipermeable membrane filters were inserted into the 24-well plates with an equal number of the chemotherapy drug-treated or -untreated cells in the bottom of the wells, and the SKOV-3 or A2780 cells (10^4^ cells/200 μL) in medium containing 1% FBS were seeded into the chambers. After 24 h of incubation, the cells in the upper surface of the chambers were removed by cotton swabs. The cells on the lower surface of the membranes were fixed and stained by 0.1% crystal violet for 15–20 min at room temperature. Rinsed with PBS twice, the stained cells were then photographed under the microscope. Finally, the cells were solubilised with 33% acetic acid (500 μl per well), and then quantified at the absorbance of 570 nm. A minimum of three wells were used for each group. All experiments were performed in triplicate.

### Wound-healing assay

Wound-healing assay was carried out as previously described.^22,23^ Afterwards, the cell-free wound areas were scratched by 10-μL sterile pipette tips. Debris was removed by rinsing with PBS, and then the culture medium was replaced with conditioned medium of the chemotherapy drug-treated or -untreated cells; in the meantime, GANT61 (Cat#S8075, Selleck Chemicals, USA) was added into the relative groups. Images were taken at 0 and 24 h after the scratch. The wound healing = (0-h width – 24-h width)/0-h width × 100%.

### Sphere formation assay

The chemotherapy drug-treated or -untreated cells were collected for sphere formation assays. GANT61 at a final concentration of 5 μM was added simultaneously into the culture medium as the cells were seeded in the relative groups. Sphere formation assays were performed as previously described with minor modifications.^24,25^ Single-cell suspensions (5 × 10^3^ cells per well) were seeded into serum-free DMEM/F12 medium (Cat# C11320033, Gibco, USA) supplemented with 20 ng/mL Animal-Free Recombinant Human EGF (EGF, Cat#AF-100-15, Peprotech, USA), 10 ng/mL Recombinant Human fibroblast growth factor-basic (bFGF, Cat#100-18B, Peprotech, USA) and B-27 supplement (Cat# 17504044, Gibco, USA) in ultra-low-attachment plates (Cat#3471, Corning, USA) and cultured for 10 days. The number of spheres formed under three different fields were evaluated under microscopy. Colony diameters >50 μm were counted as a single positive colony. For all sphere formation experiments, three wells were run for each group. All experiments were performed in triplicate.

### Flow cytometric analysis

The chemotherapy drug-treated or -untreated cells were collected for flow cytometric analysis. The flow cytometry analysis was performed as described previously.^26^ In total, 10^6^ cells in 100 μL of PBS were incubated with APC anti-human CD133 antibody (5 μL/10^6^ cells) (clone 7, Cat#372806, Biolegend, USA), FITC anti-mouse/human CD44 antibody (5 μL/10^6^ cells) (clone IM7, Cat#103006, Biolegend, USA) for 30 min in the dark at 4 °C, following the manufacturer’s instructions. Then the cells were washed with PBS twice and resuspended in 300 μL of PBS prior to FACS analysis.

### Bioinformatics analysis

Dataset GSE109934 in Gene Expression Omnibus (GEO) database (http://www.ncbi.nlm.nih.gov/geo)^27^ was used to identify differentially expressed genes (DEGs). The dataset included 19 chemotherapy samples and 19 non-chemotherapy samples of ovarian cancer patients. Microarray-based bioinformatics analysis was performed using R software and Bioconductor 3.3.2 (http://www.bioconductor.org/). We screened the significant DEGs by Limma package of R software. The fold change > 2 and *p* < 0.05 were considered statistically significant. Biological significance and pathway of DEGs was explored by GO term and KEGG enrichment analysis using ClusterProfiler package of R software. *p* < 0.05 was considered to be significant. Using the PROGgeneV2 website tool (http://watson.compbio.iupui.edu/chirayu/proggene/database/?url=proggene),^28^ the relationship between GLI1 expression and survival probability was investigated in ovarian cancer datasets by calculating *p*-value and hazard ratios (HR) with relative confidence intervals (CI).

### Quantitative real-time PCR

The quantitative real-time PCR was performed as described previously.^29^ Total RNA was extracted using Trizol (Cat#15596026, Invitrogen, USA), and complementary DNA was synthesised using TransScript First-Strand cDNA Synthesis SuperMix (Cat#AT301, TransGen Biotech, China). Specific quantitative real-time PCR experiments were performed using FastStart Universal SYBR Green Master (Rox) (Cat#4913914001, Roche, Swiss) following the manufacturer’s protocols. Specific primers used for qRT-PCR assays are listed in Table S1.

### Clinical samples

The ovarian cancer tissue samples were collected from Jilin Cancer Hospital, China. Tissue samples from patients who underwent surgery without chemotherapy (non-chemotherapy group, stage I, *n* = 6) or from patients who received chemotherapy before surgery (chemotherapy group, *n* = 16) were analysed by immunohistochemistry. The FIGO stage and histology of the tissue samples are listed in Table S2. The work was approved by the Ethics Committee of Jilin Cancer Hospital and performed in accordance with the Declaration of Helsinki. All patients signed written informed consent.

### Immunohistochemistry

Histological sections of paraffin-embedded biopsies were stained by immunohistochemistry using the UltraSensitive SP IHC Kit (Cat#KIT9710, Maxim Biotechnologies, China), following the manufacturer’s instructions. The primary antibody anti-GLI1 antibody (Cat. # ab151796, Abcam) was diluted at 1:100. Immunochemistry results were evaluated semi-quantitatively using a H score as previously described.^30^ Briefly, five fields were randomly selected. The percentage of positive cells was determined by the staining intensity (0: no signal, 0%; 1: weak signal, ≤25%; 2: intermediate signal, 25–50%; 3: strong signal, ≥50%). The H scores of each field were determined using the following formula: H score % = 1 × (% of cells with an intensity 1) + 2 × (% of cells with an intensity 2) + 3 × (% of cells with an intensity 3). Then the average of the H scores of five individual fields was calculated as the final H score of each group.

### Western blot analysis

The chemotherapy drug-treated or -untreated SKOV-3 or A2780 cells were collected for Western Blot analysis. Western blot analysis was performed as previously described.^20^ The primary antibodies, including anti-GLI1 antibody (Cat. # ab151796, Abcam), anti-Bmi 1 (D42B3) antibody (Cat. # 5856, Cell Signaling Technology) and beta-actin antibody (Cat. # 60008-1-lg, Proteintech Group, USA) were 1:1000 diluted. Secondary antibodies including HRP-conjugated Affinipure Goat Anti-Mouse IgG(H + L) (Cat. # SA00001-2, Proteintech Group, USA) and HRP-conjugated Affinipure Goat Anti-Rabbit IgG(H + L) (Cat. # SA00001-1, Proteintech Group, USA) were 1:5000 diluted.

### RNA interference

GLI1 siRNA (5’-GCGUGAGCCUGAAUCUGUGTT-3’, 5’-CACAGAUUCAGGCUCACGCTT-3’)^31^ was designed and chemically synthesised by Sangon (Shanghai, China). Scramble siRNA was used as a negative control. siRNA transfection was performed as previously described.^21^ Briefly, the cells were seeded into six-well plates and cultured for 24 h until the cells reached ~70% confluence. Then the cells were starved for 1 h in serumfree culture. The transfection mixture containing Lipofectamine 2000 (Invitrogen, Cat#11668019, USA) and the siRNA of GLI1 (40 nM) was incubated together for 20 min and then added into the culture medium gently. Cells were incubated in the mixture for 4 h, and then the medium was replaced by normal 10% FBS- containing medium. After 48 h of incubation, the expression of GLI1 was detected by real-time quantitative PCR and western blot. About 24 h after the transfection, cells were collected and seeded into 8.0-μm pore-size Transwell chambers for migration assay.

### Statistical analysis

Statistical analysis was performed by GraphPad Prism software 5. The results were presented as the mean ± SD unless indicated otherwise. The significance of difference between groups was assessed by the Student’s *t* test for single comparisons or the analysis of variance (ANOVA) with the Newman–Keuls tests for multiple comparisons. A value of *p* < 0.05 was considered statistically significant.

## Results

### Chemotherapy exacerbated the migration of ovarian cancer cell lines

Metastasis is considered as one of the major causes of cancer treatment failure. Our previous study found that chemotherapy-treated apoptotic ovarian cancer cells could induce the repopulation of a small number of surviving cells through the increased PGE2 level in the tumour microenvironment.^20^ In our study, a similar Transwell system was established as previously to explore the influence of chemotherapy-induced apoptotic ovarian cancer cells on the migration ability of the remaining ovarian cancer cells.

**Fig. 1:**
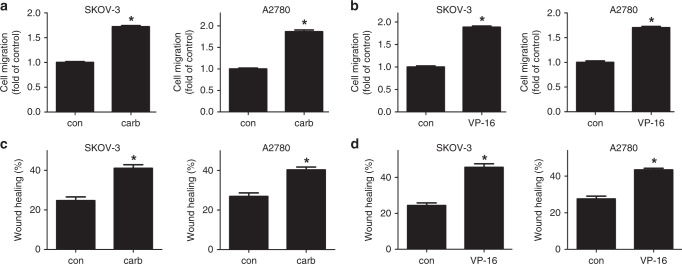
Chemotherapy exacerbated the migration of ovarian cancer cell lines. SKOV-3 and A2780 cells were treated with carboplatin or VP-16 for 24 h. The two cell lines treated with carboplatin were cultured for an extra duration of 72 h. The two cell lines treated with VP-16 were cultured for another 5–6 days. **a**, **b** Transwell migration assay was then conducted using the two cell lines respectively in the conditioned medium of the chemotherapy-treated cells. The cells on the lower surface of the semipermeable membranes were fixed and stained with 0.1% crystal violet, then solubilised with 33% acetic acid and quantified at absorbance of 570 nm. **c**, **d** Conditioned medium of the carboplatin- or VP-16-treated cell lines was used in the wound-healing assay of the SKOV-3 and A2780 cell lines. The results were expressed as the mean ± SD, *p < 0.05 compared with the control group.

Two representative ovarian cancer cell lines SKOV-3 and A2780 were used. A first-line chemotherapy drug carboplatin and a second-line chemotherapy drug VP-16 was used respectively as chemotherapy treatments. Compared with vehicle treatment, the 24-h treatments of carboplatin or VP-16 significantly induced the death of feeder cells (Fig. S1). The altered microenvironment of either carboplatin- or VP-16-treated ovarian cancer cells significantly increased the migration of the two cell lines (Fig. 1a–d, Fig. S2A–D). This chemotherapy- exacerbated migration was also observed in the KURAMOCHI cell line that was reported to be most similar to the high-grade serous ovarian cancer (HGSOC) cells^32^ (Fig. S3A–C).

### Chemotherapy induced the cancer stem cell (CSC)-like characteristics of ovarian cancer cell lines

**Fig. 2:**
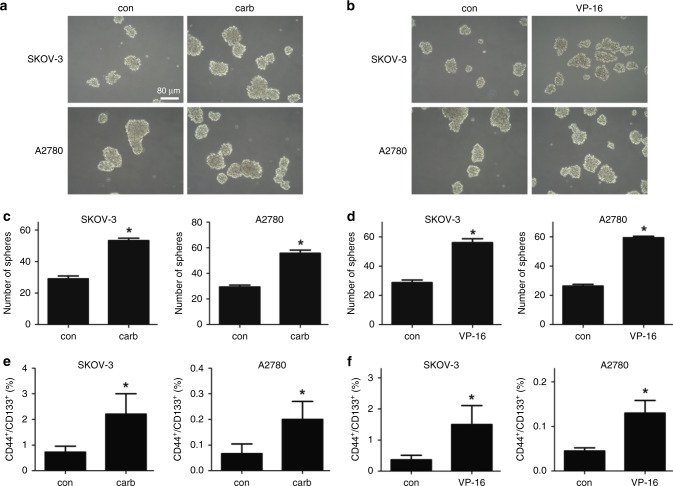
Chemotherapy induced the cancer stem cell (CSC)-like characteristics of ovarian cancer cell lines. **a**, **b** The SKOV-3 and A2780 cell lines co-cultured with the carboplatin- or VP-16-treated cells were used in the sphere formation assay and cultured for 10 days. Colony diameters >50 μm were counted as a single positive colony. **c**, **d** Quantitative analysis of positive colonies in the sphere formation assay. **e**, **f** The expression of the OCSC markers (CD44+/CD133+) in the SKOV-3 and A2780 cell lines co-cultured with the carboplatin- or VP-16-treated cells was analysed by flow cytometric analysis. All experiments were performed in triplicate. The results were expressed as the mean ± SD of three parallel experiments, *p < 0.05 compared with the control group.

**Fig. 3:**
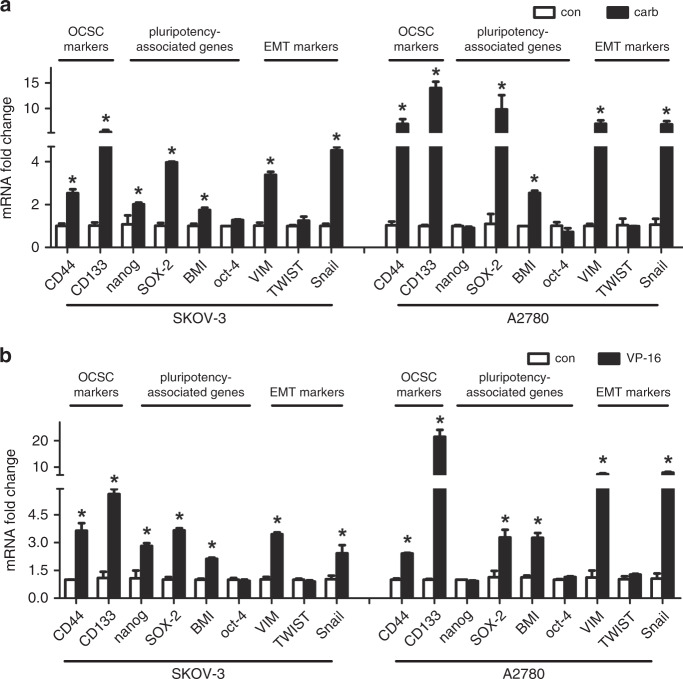
Expression of the pluripotency-associated genes, OCSC markers and EMT markers after the chemotherapy treatments. **a**, **b** The SKOV-3 and A2780 cell lines co-cultured with carboplatin- or VP-16-treated cells were analysed by qRT-PCR. The mRNA expression of the reported cancer stem cell markers, pluripotency-associated genes and EMT markers in ovarian cancer was investigated. All experiments were performed in triplicate. The results were expressed as the mean ± SD, *p < 0.05 compared with the control group.

Since studies have shown that the migration ability of tumour cells was associated with the cancer stem cell-like properties, we studied the influence of chemotherapy treatment on the CSC-like properties of the two ovarian cancer cell lines. Ovarian cancer cells co-cultured with either carboplatin- or VP-16-treated cells exhibited higher sphere formation ability (Fig. 2a–d), which was also confirmed in the KURAMOCHI cell line (Fig. S3D, E). The properties of these cells expressing the OCSC markers (CD44+/CD133+) (Fig. 2e, f) and the mRNA level of CD44 and CD133 were also significantly increased in the microenvironment of chemotherapy-challenged cells (Fig. 3a, b), which confirmed again that the chemotherapy treatment can significantly increase the CSC properties of ovarian cancer cells. The expression of three EMT markers in ovarian cancer was also investigated. The expression of Vimentin (VIM) and Snail was significantly increased, while the expression of Twist remained still (Fig. 3a, b). Then the expression of several reported key genes that may regulate the CSC-like characteristics was analysed in the receptor cells. The expression of SOX-2 and BMI was dramatically increased in both cell lines treated by both drugs. The expression of Nanog was significantly increased in SKOV-3 cells but not in A2780 cells, suggesting that the expression of Nanog may differ in different cell lines. The expression of Oct-4 was not regulated by either chemotherapy treatment in both cell lines (Fig. 3a, b). Therefore, we focus on the two pluripotency-associated genes SOX-2 and BMI to investigate the influence of chemotherapy on them.

### The chemotherapy-induced cancer stem cell-like property increase is associated with transcription factor GLI1

**Fig. 4:**
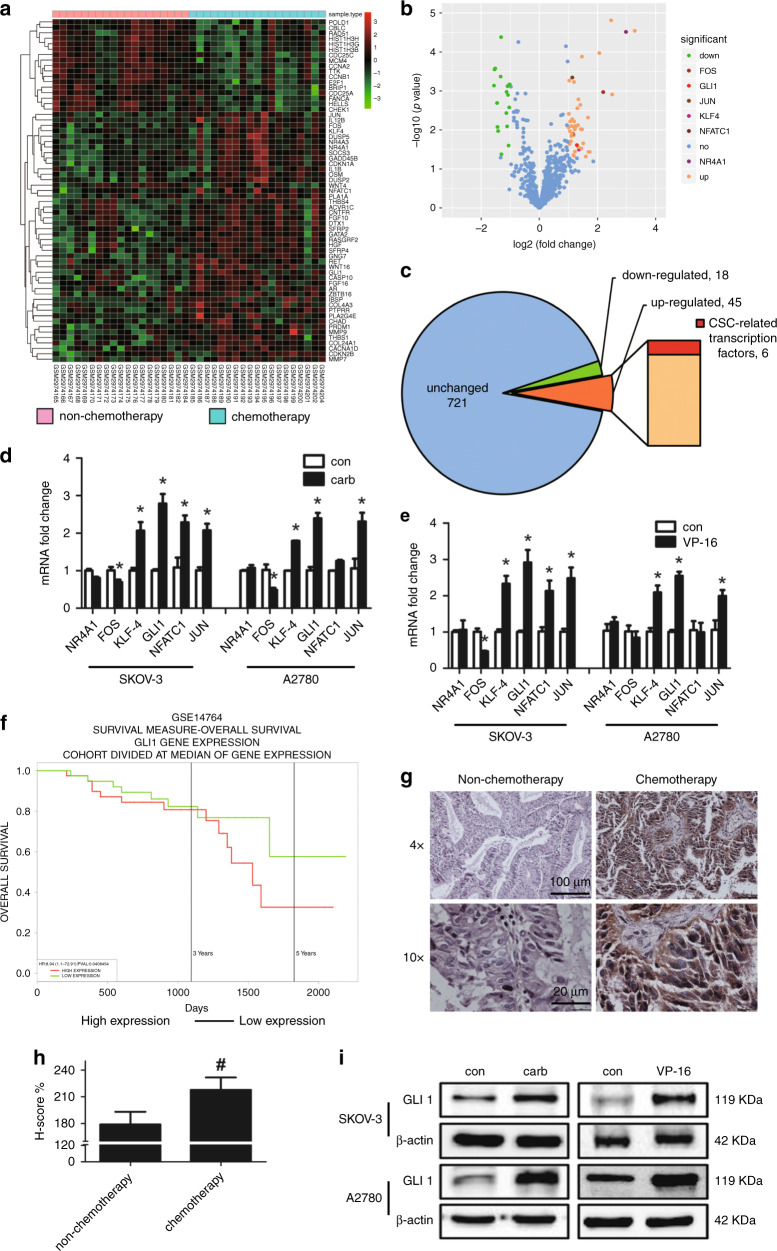
The chemotherapy-exacerbated cancer stem cell-like property increase is associated with the expression of GLI1. **a**, **b** Dataset GSE109934 in GEO database (http://www.ncbi.nlm.nih.gov/geo), including 19 chemotherapy samples and 19 non-chemotherapy samples of ovarian cancer patients, was used to identify DEGs. **c** The expression of 784 genes in the gene chips of the above ovarian cancer tissues was analysed. In total, 45 upregulated (orange ones) and 18 downregulated genes (green ones) were identified. Among them, six transcription factors regulating the cancer stem cell-associated genes were verified by the reported literatures. **d**, **e** The SKOV-3 and A2780 cell lines co-cultured with the carboplatin- or VP-16-treated cells were analysed by qRT-PCR. The six reported transcription factors regulating the cancer stem cell-associated genes were investigated. **f** The relationship between GLI1 expression and survival probability was investigated in ovarian cancer datasets by the PROGgeneV2 website tool (http://watson.compbio.iupui.edu/chirayu/proggene/database/?url=proggene). **g** Immunohistochemistry analysis of tissue samples from patients who underwent surgery without chemotherapy (control group, *n* = 6) or from patients who received chemotherapy before surgery (chemotherapy group, *n* = 16). **h** Quantitative analysis of the IHC analysis. **i** After the 3–5 days’ co-culture, the receptor cells co-cultured with the carboplatin- or VP-16-treated feeder cells were collected for western blot analysis. The results were expressed as the mean ± SD, *p < 0.05 compared with the control group. ^#^*p* < 0.05 compared with the non-chemotherapy group.

In order to identify the potential transcription factors that were alternated by chemotherapy treatment and may regulate the expression of pluripotency-associated genes, the expression of 784 genes in the gene chips of ovarian cancer tissues treated or untreated by chemotherapeutics was analysed. By using Limma package of R software, a total of 63 DEGs were identified between the chemotherapy and non-chemotherapy groups in ovarian cancer patients (Fig. 4a). Hierarchical clustering analysis was obtained for the 63 DEGs. The general gene expression patterns were evidently different in the two groups (chemotherapy vs. non-chemotherapy) presented by TreeView, including 45 upregulated (orange ones) and 18 downregulated genes (green ones, Fig. 4b). Among them, six transcription factors regulating the cancer stem cell-associated genes were verified by the reported literatures (marked with deep red, Fig. 4b, c), including NR4A1, FOS, KLF-4, GLI1, NFATC1 and JUN (Table S3).

Based on this, the qRT-PCR was conducted to verify the alternations of these genes in the two cell lines treated by the two different chemotherapy drugs. GLI1 was confirmed to be upregulated by either carboplatin or VP-16 treatments in both cell lines most significantly (Fig. 4d, e). KEGG and GO analysis was done to analyse the biological significance and pathways of the DEGs. In KEGG analysis, the DEGs were mainly enriched in pathways in cancer, cell cycle, MAPK signalling pathway, Wnt signalling pathway and p53 signalling pathway. GLI1 was enriched in pathways in cancer that was the most significant pathway (Fig. S4A). In GO analysis, the significant biological functions of GLI1 were regulation of cell proliferation, positive regulation of macromolecule metabolic process and positive regulation of macromolecule biosynthetic process (Fig. S4B). Survival analysis was performed using the PROGgeneV2 database, to explore the relationship between the expression of GLI1 and overall survival of ovarian cancer samples. The results suggested that 5-year overall survival was significantly decreased in the GLI1 highly expressed group. The high expression of GLI1 indicated a poor prognosis (Fig. 4f). The expression of GLI1 in clinical samples from ovarian cancer patients who went through chemotherapy (chemotherapy group) or not (non-chemotherapy group) was also analysed (Fig. 4g, h). The GLI1 expression of the chemotherapy group significantly increased compared with the non-chemotherapy group. Western blot of the chemotherapy drug-treated ovarian cancer cell lines showed similar results. The expression of GLI1 of the receptor cells was significantly upregulated in chemotherapy groups (Fig. 4i), also confirmed in KURAMOCHI cells (Fig. S3F). These indicated that GLI1 as a key transcription factor in the Hedgehog signalling pathway may play a crucial role in the chemotherapy-induced regulation of cancer stem cell-like properties.

### Downregulation of GLI1 inhibited the chemotherapy-exacerbated CSC-like properties and migration in ovarian cancer cell lines

**Fig. 5:**
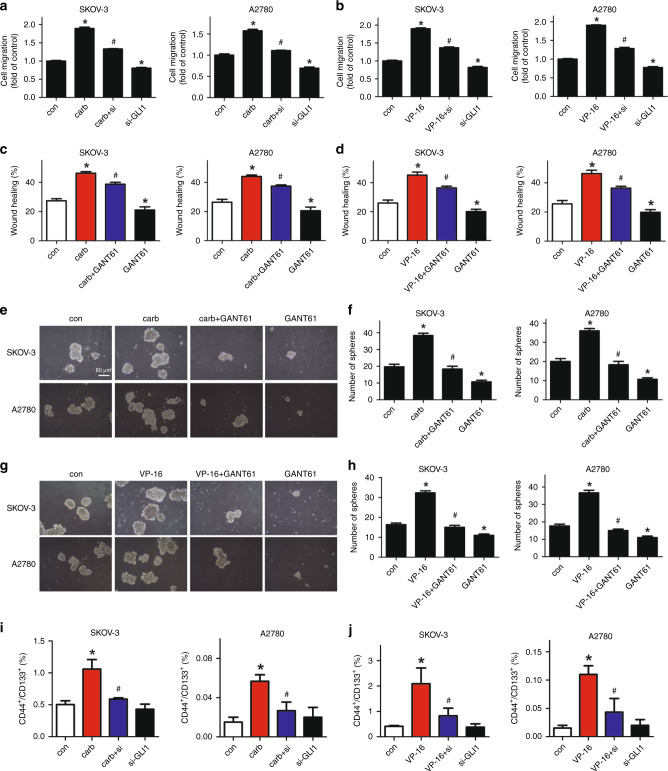
Downregulating GLI1 inhibited the chemotherapy-exacerbated CSC-like property increase and migration in ovarian cancer cells. **a**, **b** SKOV-3 or A2780 cells were transfected with negative control or siRNA of GLI1 for 24 h. Then the cells were seeded into 8.0-μm pore-size Transwell chambers in 24-well plates with chemotherapy drug-treated or -untreated cells in the bottom of the wells for migration assay. **c**, **d** The conditioned medium of the carboplatin- or VP-16-treated cells was used in the wound-healing assay. GANT61 was added simultaneously with the replacement of the conditioned culture medium. **e**–**h** The chemotherapy drug-treated or -untreated cells were collected and reseeded into the ultra-low- attachment plates and cultured for 10 days for sphere formation assays. GANT61 was added simultaneously into the culture medium as the cells were seeded in the relative groups. **i**, **j** The receptor cells transfected with negative control or siRNA of GLI1 were co-cultured with the chemotherapy drug-treated or -untreated cells (feeder cells) for 72 h and then collected for FACS analysis. All experiments were performed in triplicate. The results were expressed as the mean ± SD, *p < 0.05 compared with the control group. ^#^*p* < 0.05 compared with carb or VP-16 group.

To explore the role GLI1 may play in regulating the chemotherapy-exacerbated CSC-like properties and migration, siRNA was used to knock down the GLI1 expression (Fig. S5). As shown in Fig. 5a, b and Fig. S6A, the migration induced by carboplatin (carb group) or VP-16 treatments (VP-16 group) was significantly reversed by knocking down the expression of GLI1 in both cell lines (VP-16 + si group or carb + si group). The siRNA of GLI1 (si-GLI1 group) could also inhibit the migration of both cell lines compared with the NC-treated ones (control group). The inhibitor of GLI1 GANT61 was also used in the wound-healing assay; similar results were observed in both cell lines treated with carboplatin or VP-16 (Figs. 5c, d, S6B). The expression of GLI1 was inhibited by GANT61 in both cell lines (Fig. S7). The sphere formation ability enhanced by the chemotherapy treatments (carb group or VP-16 group) was also dramatically inhibited by GANT61 (carb + GANT61 group or VP-16 + GANT61 group) (Fig. 5e–h). Flow cytometric analysis showed that the increased cancer stem cell-like properties (CD44+/CD133+) after chemotherapy treatments (carb group or VP-16 group) were reversed by knocking down GLI1 (VP-16 + si group or carb + si group) (Fig. 5i, j), indicating the crucial role of GLI1 in the regulation of CSC-like characteristics.

### Chemotherapy exacerbated the CSC-like properties of ovarian cancer cell lines through GLI1–BMI1 signalling pathway

**Fig. 6:**
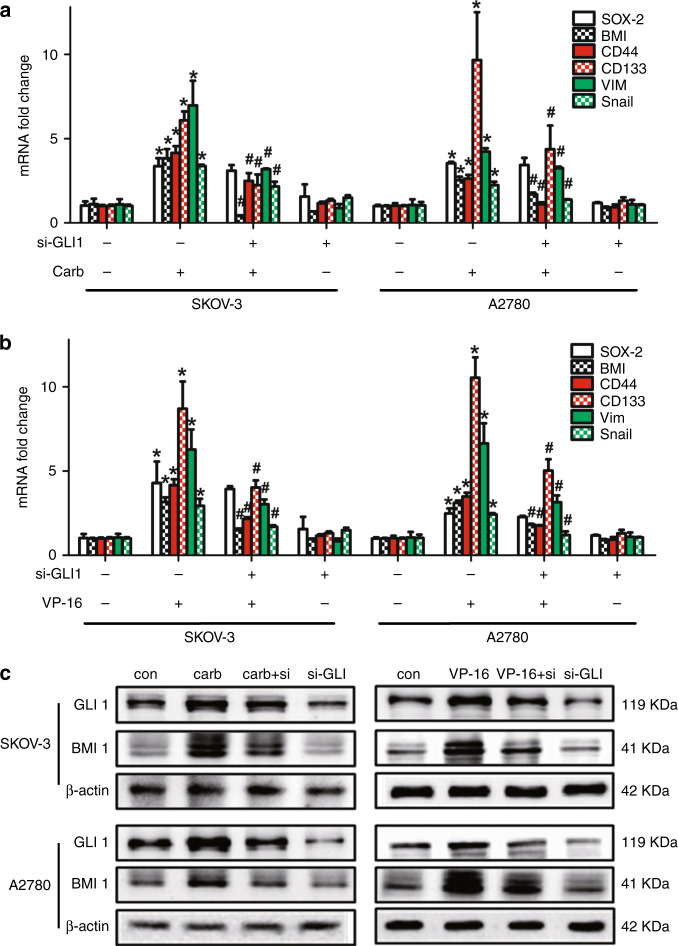
Chemotherapy induced the CSC-like properties of ovarian cancer cell lines through GLI1–BMI1 signalling pathway. The siRNA or NC-transfected receptor cells were co-cultured with chemotherapy-treated or -untreated feeder cells. **a**, **b** After 48 h of co-culture, the key genes that were observed to be regulated by the chemotherapy treatments in the receptor cells were tested by qRT-PCR. **c** After 72 h of co-culture, the receptor cells were collected for western blot analysis. All experiments were performed in triplicate. The results were expressed as the mean ± SD, *p < 0.05 compared with si-GLI^−^/carb^−^ or VP-16^−^ group. ^#^*p* < 0.05 compared with si-GLI^−^/carb^+^ or VP-16^+^ group.

The key genes that were observed to be regulated by the chemotherapy treatments were then tested by qRT-PCR to investigate whether they were regulated by the transcription factor GLI1. It was shown that although the two pluripotency-associated genes SOX-2 and BMI were significantly upregulated by chemotherapy treatments, silencing GLI1 did not regulate the enhanced SOX-2 expression, but significantly inhibited the chemotherapy-induced upregulation of BMI1 in the receptor cells of both chemotherapy-challenged groups. Therefore, the upregulation of BMI1 but not SOX-2 may be regulated by GLI1. The expression of the chemotherapy-induced upregulated EMT markers (VIM and Snail) and stem cell markers (CD44 and CD133) in ovarian cancer was also reserved by GLI1 knockdown (Fig. 6a, b). The protein expression of GLI1 and BMI was also upregulated in the receptor cells of both chemotherapy-challenged groups in both cell lines, while GLI1 silencing could reverse this upregulation (Fig. 6c). These results indicated that the GLI1–BMI1 signalling pathway may be one of the activated pathways in the process of chemotherapy-exacerbated migration and the increase in CSC-like characteristics.

## Discussion

It is becoming clear that ovarian cancer is a heterogeneous disease.^33^ Approximately 90% of the ovarian cancer is epithelial cancer. Other subtypes include germ cell (2.6%), sex-cord stromal (1.2%) and mixed-cell types. Among the epithelial ovarian cancer subtypes, 68–71% patients were classified as having serous histology. Other histological subtypes include endometrioid (9–11%), clear cell (12–13%), mucinous (3%), malignant Brenner tumours (1%) and mixed histologies (6%).^34,35^ Histologic and molecular characters differ in different histological types.^36^ In our study, two mostly used ovarian cancer cell lines SKOV-3 and A2780, as well as KURAMOCHI cell line that is most genomically similar to the high-grade serous ovarian cancer (HGSOC) cells,^32^ were employed to explore the unexpected influence of chemotherapy on migration of ovarian cancer cells remaining in vitro rather than the widely known therapeutic efficacy. SKOV-3 cells were collected from the ascites of an ovarian cancer patient and represent well-differentiated serous adenocarcinoma cancer. This cell line forms xenograft tumours with a histology very similar to a human serous cancer.^37^ A2780 cells were collected from the tumour tissue with no clear histological classification in the original publication. The putative histological subtype was endometrioid or clear cell.^38^ Our results first indicated that all the three cell lines showed similar response to the first- or second-line chemotherapy treatments: the increase in CSC-like characteristics and migration. It first indicated that the chemotherapy-exacerbated metastasis may be universal in the treatment of different histological types of ovarian cancer. This phenomenon may be attributed to generic host repair mechanism in response to chemotherapy-induced extensive tissue damage, but does not depend on the specific types of chemotherapy agents.

Studies have described the increase in CSCs and EMT induced by chemotherapy treatments. However, whether the induction of CSCs and EMT is due to the selection of chemotherapy or mediated by chemotherapy treatments remains disunited.^4,39,40^ Among the studies demonstrating that chemotherapy may induce the CSC-like traits or the migration ability of tumour cells, whether chemotherapy directly triggers intracellular signalling or it may alter the microenvironment while killing the tumour cells and indirectly influence the EMT of the remaining cells or the two ways exist also remains unclear.^4,8,14^ In our study, the Transwell co-culture system was established to solely investigate the influence of the altered microenvironment by chemotherapy-treated dying cells on the tumour cells. We proved that chemotherapy-treated dying cells altered the microenvironment and induced the CSC-like characteristics by upregulating the GLI1–BMI1 pathway.

The chemotherapy-altered factors in the tumour microenvironment that lead to the upregulation of GLI1 may be different. Our previous study has observed the increased level of PGE2 in the microenvironment of chemotherapy-induced apoptotic cells, which leads to the proliferation of ovarian cancer cells.^20,41^ PGE2 is known as an important inducer of the proliferation and migration of cancers,^42^ and it was reported to activate GLI1 by the activation of integrin β1.^43^ Another recent study has indirectly co-cultured cancer cells and bone marrow mesenchymal stem cells (BM-MSCs) without direct cell contact: a paracrine effect of cancer cells has been observed by secreting soluble factors, such as TGF-β, which promoted a more stem-like state of non-CSCs.^44^ In line with these researches, our study emphasised the role of microenvironment in the chemotherapy-induced CSC-like traits, and the upregulation of GLI1 may result from several different factors in the chemotherapy-altered tumour microenvironment.

Recent studies have shown steady progress in demonstrating the mechanism of chemotherapy-exacerbated cancer metastasis: paclitaxel-involved neoadjuvant chemotherapy was proven to induce breast cancer metastasis in a tumour microenvironment of metastasis (TMEM)-mediated way.^6,7^ However, the randomised controlled or prospective clinical studies investigating the influence of chemotherapy on the metastasis of ovarian cancer are limited due to the present treatment guideline and the difficulty in obtaining the cases of early-stage patients. To investigate the possible impact of chemotherapy on the metastatic potential of ovarian cancer, information of 163 consecutive ovarian cancer patients between August 2018 and May 2019 at Jilin Cancer Hospital, Jilin, China was collected. Among all patients who underwent chemotherapy, patients without metastasis at the first diagnosis (FIGO stage Ia and Ib) and whose stages of metastasis were again confirmed by open exploration in 5 years were included for the analysis. Due to the extremely low diagnosis rate of early-stage ovarian cancer, only four patients met the inclusion criteria. Three of them had intra-abdominal, lymphatic or distant metastasis in the second open exploration. One patient accepted secondary operation due to the recurrence of ovarian cancer and no metastasis was found. Despite the difficulty in finding the proper cases due to the characteristics of ovarian cancer, there may be a possibility that in non-metastasis patients who underwent chemotherapy, the primary tumour tends to metastasise. Also, in our study, there was significant difference on the stages and histology in our tissue samples with or without chemotherapy, and it was extremely difficult to be controlled for. Therefore, there may be other factors such as stages and histology that contributed to the higher expression of GLI1 in the chemotherapy group. More efforts are still needed to fully explore the possible impact of chemotherapy on the metastatic potential of ovarian cancer.

In conclusion, chemotherapy may trigger the induction of CSC-like characteristics and metastasis of ovarian cancer while killing the tumour cells by GLI1–BMI1 signalling. The inhibition of GLI1 following chemotherapy may serve as a novel strategy to ensure the crucial killing effect of chemotherapy on tumour cells and inhibit the chemotherapy-exacerbated metastasis in ovarian cancer treatment.

## Supplementary information

western-blot raw data

Supplementary Materials

## Data Availability

The datasets used and/or analysed during this study are available from the corresponding author on reasonable request. The RNA-seq dataset GSE109934 is available in the Gene Expression Omnibus (https://www.ncbi.nlm.nih.gov/geo/).^27^ The survival analysis was performed using the PROGgeneV2 website tool (http://watson.compbio.iupui.edu/chirayu/proggene/database/?url=proggene).^28^

## References

[1] Lengyel E (2010). Ovarian cancer development and metastasis. Am. J. Pathol..

[2] Cannistra SA (2004). Cancer of the ovary. N. Engl. J. Med..

[3] Salani R, Backes FJ, Fung MF, Holschneider CH, Parker LP, Bristow RE (2011). Posttreatment surveillance and diagnosis of recurrence in women with gynecologic malignancies: Society of Gynecologic Oncologists recommendations. Am. J. Obstet. Gynecol..

[4] Karagiannis GS, Condeelis JS, Oktay MH (2018). Chemotherapy-induced metastasis: mechanisms and translational opportunities. Clin. Exp. metastasis.

[5] Wu YJ, Muldoon LL, Dickey DT, Lewin SJ, Varallyay CG, Neuwelt EA (2009). Cyclophosphamide enhances human tumor growth in nude rat xenografted tumor models. Neoplasia.

[6] Chang YS, Jalgaonkar SP, Middleton JD, Hai T (2017). Stress-inducible gene Atf3 in the noncancer host cells contributes to chemotherapy-exacerbated breast cancer metastasis. Proc. Natl Acad. Sci. USA.

[7] Karagiannis George S., Pastoriza Jessica M., Wang Yarong, Harney Allison S., Entenberg David, Pignatelli Jeanine, Sharma Ved P., Xue Emily A., Cheng Esther, D’Alfonso Timothy M., Jones Joan G., Anampa Jesus, Rohan Thomas E., Sparano Joseph A., Condeelis John S., Oktay Maja H. (2017). Neoadjuvant chemotherapy induces breast cancer metastasis through a TMEM-mediated mechanism. Science Translational Medicine.

[8] Liu G, Chen Y, Qi F, Jia L, Lu XA, He T (2015). Specific chemotherapeutic agents induce metastatic behaviour through stromal- and tumour-derived cytokine and angiogenic factor signalling. J. Pathol..

[9] McLeary F, Davis A, Rudrawar S, Perkins A, Anoopkumar-Dukie S (2019). Mechanisms underlying select chemotherapeutic-agent-induced neuroinflammation and subsequent neurodegeneration. Eur. J. Pharmacol..

[10] Wang Y, Cardenas H, Fang F, Condello S, Taverna P, Segar M (2014). Epigenetic targeting of ovarian cancer stem cells. Cancer Res..

[11] Burgos-Ojeda D, Rueda BR, Buckanovich RJ (2012). Ovarian cancer stem cell markers: prognostic and therapeutic implications. Cancer Lett..

[12] Gonzalez DM, Medici D (2014). Signaling mechanisms of the epithelial-mesenchymal transition. Sci. Signal..

[13] Dasgupta A, Sawant MA, Kavishwar G, Lavhale M, Sitasawad S (2016). AECHL-1 targets breast cancer progression via inhibition of metastasis, prevention of EMT and suppression of cancer stem cell characteristics. Sci. Rep..

[14] Abubaker K, Latifi A, Luwor R, Nazaretian S, Zhu H, Quinn MA (2013). Short-term single treatment of chemotherapy results in the enrichment of ovarian cancer stem cell-like cells leading to an increased tumor burden. Mol. cancer.

[15] Yanai K, Nagai S, Wada J, Yamanaka N, Nakamura M, Torata N (2007). Hedgehog signaling pathway is a possible therapeutic target for gastric cancer. J. Surgical Oncol..

[16] Yu D, Shin HS, Lee YS, Lee D, Kim S, Lee YC (2014). Genistein attenuates cancer stem cell characteristics in gastric cancer through the downregulation of Gli1. Oncol. Rep..

[17] Clement V, Sanchez P, de Tribolet N, Radovanovic I, Ruiz i, Altaba A (2007). HEDGEHOG-GLI1 signaling regulates human glioma growth, cancer stem cell self-renewal, and tumorigenicity. Curr. Biol..

[18] Fu J, Rodova M, Roy SK, Sharma J, Singh KP, Srivastava RK (2013). GANT-61 inhibits pancreatic cancer stem cell growth in vitro and in NOD/SCID/IL2R gamma null mice xenograft. Cancer Lett..

[19] Sun Y, Wang Y, Fan C, Gao P, Wang X, Wei G (2014). Estrogen promotes stemness and invasiveness of ER-positive breast cancer cells through Gli1 activation. Mol. Cancer.

[20] Zhao Yawei, Cui Lianzhi, Pan Yue, Shao Dan, Zheng Xiao, Zhang Fan, Zhang Hansi, He Kan, Chen Li (2017). Berberine inhibits the chemotherapy-induced repopulation by suppressing the arachidonic acid metabolic pathway and phosphorylation of FAK in ovarian cancer. Cell Proliferation.

[21] Yang N, Hui L, Wang Y, Yang H, Jiang X (2014). Overexpression of SOX2 promotes migration, invasion, and epithelial-mesenchymal transition through the Wnt/beta-catenin pathway in laryngeal cancer Hep-2 cells. Tumour Biol..

[22] Liao J, Qian F, Tchabo N, Mhawech-Fauceglia P, Beck A, Qian Z (2014). Ovarian cancer spheroid cells with stem cell-like properties contribute to tumor generation, metastasis and chemotherapy resistance through hypoxia-resistant metabolism. PLoS ONE.

[23] Chen D, Cao G, Qiao C, Liu G, Zhou H, Liu Q (2018). Alpha B-crystallin promotes the invasion and metastasis of gastric cancer via NF-kappaB-induced epithelial-mesenchymal transition. J. Cell. Mol. Med..

[24] Young MJ, Wu YH, Chiu WT, Weng TY, Huang YF, Chou CY (2015). All-trans retinoic acid downregulates ALDH1-mediated stemness and inhibits tumour formation in ovarian cancer cells. Carcinogenesis.

[25] Lin J, Zhang L, Huang H, Huang Y, Huang L, Wang J (2015). MiR-26b/KPNA2 axis inhibits epithelial ovarian carcinoma proliferation and metastasis through downregulating OCT4. Oncotarget.

[26] Zhao Y, Jia Y, Shi T, Wang W, Shao D, Zheng X (2019). Depression promotes hepatocellular carcinoma progression through a glucocorticoids Mediated up-regulation of PD-1 expression in tumor infiltrating NK Cells. Carcinogenesis.

[27] Edgar R, Domrachev M, Lash AE (2002). Gene Expression Omnibus: NCBI gene expression and hybridization array data repository. Nucleic Acids Res..

[28] Goswami CP, Nakshatri H (2014). PROGgeneV2: enhancements on the existing database. BMC Cancer.

[29] Bian XL, Chen HZ, Yang PB, Li YP, Zhang FN, Zhang JY (2017). Nur77 suppresses hepatocellular carcinoma via switching glucose metabolism toward gluconeogenesis through attenuating phosphoenolpyruvate carboxykinase sumoylation. Nat. Commun..

[30] Hamaidi I, Coquard C, Danilin S, Dormoy V, Beraud C, Rothhut S (2019). The Lim1 oncogene as a new therapeutic target for metastatic human renal cell carcinoma. Oncogene.

[31] Feng YZ, Shiozawa T, Miyamoto T, Kashima H, Kurai M, Suzuki A (2007). Overexpression of hedgehog signaling molecules and its involvement in the proliferation of endometrial carcinoma cells. Clin. Cancer Res..

[32] Domcke S., Sinha R., Levine D. A., Sander C., Schultz N. Evaluating cell lines as tumour models by comparison of genomic profiles. *Nat. Commun.* **4**, 10.1038/ncomms3126 (2013). 10.1038/ncomms3126 PMC3715866 23839242

[33] Blagden SP (2015). Harnessing pandemonium: the clinical implications of tumor heterogeneity in ovarian cancer. Front. Oncol..

[34] McCluggage WG (2011). Morphological subtypes of ovarian carcinoma: a review with emphasis on new developments and pathogenesis. Pathology.

[35] Duska LR, Kohn EC (2017). The new classifications of ovarian, fallopian tube, and primary peritoneal cancer and their clinical implications. Ann. Oncol.: Off. J. Eur. Soc. Med. Oncol..

[36] Rojas Veronica, Hirshfield Kim, Ganesan Shridar, Rodriguez-Rodriguez Lorna (2016). Molecular Characterization of Epithelial Ovarian Cancer: Implications for Diagnosis and Treatment. International Journal of Molecular Sciences.

[37] Lengyel E, Burdette JE, Kenny HA, Matei D, Pilrose J, Haluska P (2014). Epithelial ovarian cancer experimental models. Oncogene.

[38] Beaufort CM, Helmijr JC, Piskorz AM, Hoogstraat M, Ruigrok-Ritstier K, Besselink N (2014). Ovarian cancer cell line panel (OCCP): clinical importance of in vitro morphological subtypes. PLoS ONE.

[39] Levina V, Marrangoni AM, DeMarco R, Gorelik E, Lokshin AE (2008). Drug-selected human lung cancer stem cells: cytokine network, tumorigenic and metastatic properties. PLoS ONE.

[40] Gandhi S, Chandna S (2017). Radiation-induced inflammatory cascade and its reverberating crosstalks as potential cause of post-radiotherapy second malignancies. Cancer Metastasis Rev..

[41] Cui L, Zhao Y, Pan Y, Zheng X, Shao D, Jia Y (2017). Chemotherapy induces ovarian cancer cell repopulation through the caspase 3-mediated arachidonic acid metabolic pathway. OncoTargets Ther..

[42] Li HJ, Reinhardt F, Herschman HR, Weinberg RA (2012). Cancer-stimulated mesenchymal stem cells create a carcinoma stem cell niche via prostaglandin E2 signaling. Cancer Discov..

[43] Chong Y, Tang D, Xiong Q, Jiang X, Xu C, Huang Y (2016). Galectin-1 from cancer-associated fibroblasts induces epithelial-mesenchymal transition through beta1 integrin-mediated upregulation of Gli1 in gastric cancer. J. Exp. Clin. Cancer Res..

[44] El-Badawy A, Ghoneim MA, Gabr MM, Salah RA, Mohamed IK, Amer M (2017). Cancer cell-soluble factors reprogram mesenchymal stromal cells to slow cycling, chemoresistant cells with a more stem-like state. Stem Cell Res. Ther..

