# Supplementary figures and images for: Chemotherapy exacerbates ovarian cancer cell migration and cancer stem cell-like characteristics through GLI1
Source: Br J Cancer. 2020 Apr 3;122(11):1638–48. doi: 10.1038/s41416-020-0825-7 (PMC7250874; doi:10.1038/s41416-020-0825-7)

SKOV-3

GLI1

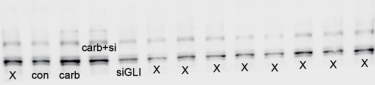

GLI1

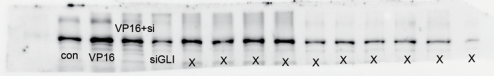

BMI1

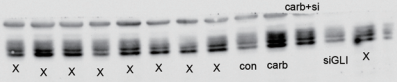

BMI1

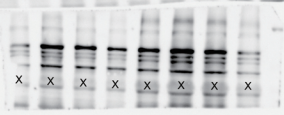

actin

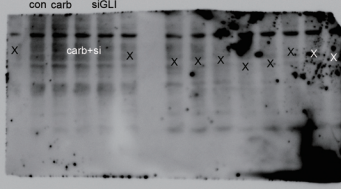

actin

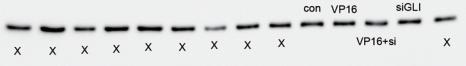

A2780

GLI1

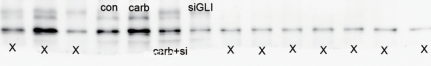

GLI1

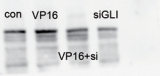

BMI1

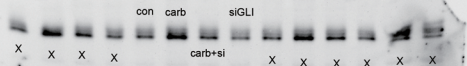

BMI1

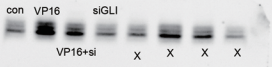

actin

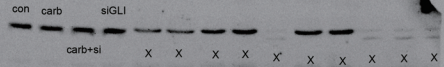

actin

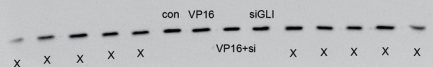

Supplement: Supplementary file 1 — western-blot raw data [file 41416_2020_825_MOESM1_ESM.pdf]
