# Supplementary material for: Chemotherapy exacerbates ovarian cancer cell migration and cancer stem cell-like characteristics through GLI1
Source: Br J Cancer. 2020 Apr 3;122(11):1638–48. doi: 10.1038/s41416-020-0825-7 (PMC7250874; doi:10.1038/s41416-020-0825-7)
Supplement: Supplementary file 2 — Supplementary Materials [file 41416_2020_825_MOESM2_ESM.docx]

**Supplementary Materials**

**Table S1. Oligonucleotides used in qRT-PCR.**

| Name |  | Sequence (5’→3’) |
| --- | --- | --- |
| qRT-PCR | | |
| *SOX-2* | Fw | GGGAAATGGGAGGGGTGCAAAAGA |
|  | Rev | TTGCGTGAGTGTGGATGGGATTGG |
| *GLI 1* | Fw | CCCAATCACAAGTCAGGTTCCT |
|  | Rev | CCTATGTGAAGCCCTATTTGCC |
| *BMI 1* | Fw | CCTTCATTGTCTTTTCCGCCC |
|  | Rev | AAGTACCCTCCACAAAGCAC |
| *VIM* | Fw | GCCTGCAGGATGAGATTCAGAATA |
|  | Rev | AACCAGAGGGAGTGAATCCAGATTA |
| *CD133* | Fw | GCATTGGCATCTTCTATGGTT |
|  | Rev | CGCCTTGTCCTTGGTAGTGT |
| *Snail* | Fw | GGAAGCCTAACTACAGCGAG |
|  | Rev | CAGAGTCCCAGATGAGCATTG |
| *Nanog* | Fw | CTCCAACATCCTGAACCTCAGC |
|  | Rev | CGTCACACCATTGCTATTCTTCG |
| *Twist* | Fw | GCCAGGTACATCGACTTCCTCT |
|  | Rev | TCCATCCTCCAGACCGAGAAGG |
| *Gapdh* | Fw | GGTGGTCTCCTCTGACTTCAACA |
|  | Rev | GTTGCTGTAGCCAAATTGCTTGT |
| *CD44* | Fw | AGAAGGTGTGGGCAGAAGAA |
|  | Rev | AAATGCACCATTTCCTGAGA |
| *Oct-4* | Fw | CCTGAAGCAGAAGAGGATCACC |
|  | Rev | AAAGCGGCAGATGGTCGTTTGG |
| *KIF4* | Fw | GTCAGTTCATCTGAGCGGG |
|  | Rev | AGAGTTCCCATCTCAAGGCA |
| *NR4A1* | Fw | CTCTGGAGGTCATCCGCAAG |
|  | Rev | CTGGCTTAGACCTGTACGCC |
| *cJUN* | Fw | TAACCTCACGTGAAGTGACG |
|  | Rev | GGCTTTAGTTCTCGGACACT |
| *cFOS* | Fw | AGTGGAACCTGTCAAGAGCAT |
|  | Rev | GCTCCCAGTCTGCTGCATAGA |
| *NFATC* | Fw | TGCAAGCCGAATTCTCTGG |
|  | Rev | GGGAAGGTAGGTGAAACGCTG |

**Table S2** FIGO Stage and Histology of tissue samples from ovarian cancer patients

| Group | No. | FIGO Stage | Histology |
| --- | --- | --- | --- |
| Non-chemotherapy | 1 | I | Clear cell |
|  | 2 | I | Mucinous |
|  | 3 | I | Serous |
|  | 4 | I | Clear cell |
|  | 5 | II | Serous |
|  | 6 | I | Granulosa cell |
| chemotherapy | 7 | IV | Mucinous |
|  | 8 | III | Serous |
|  | 9 | IV | Clear cell |
|  | 10 | IV | Serous |
|  | 11 | IV | Serous |
|  | 12 | III | Serous |
|  | 13 | III | Serous |
|  | 14 | III | Serous |
|  | 15 | IV | Serous |
|  | 16 | IV | Serous |
|  | 17 | IV | Adenocarcinoma |
|  | 18 | III | Serous |
|  | 19 | III | Serous |
|  | 20 | IV | Serous |
|  | 21 | III | Endometrioid |
|  | 22 | IV | Serous |

**Table S3.** Transcription factors related to the regulation of cancer stem cells among the DEGs between the chemotherapy and non-chemotherapy group.

|  | LogFC | p value | Regulation of CSC |
| --- | --- | --- | --- |
| NR4A1 | 2.98 | <0.0001 | ↑ |
| FOS | 2.19 | 0.001 | ↑ |
| KLF-4 | 1.36 | 0.032 | ↑ |
| GLI1 | 1.29 | 0.024 | ↑ |
| NFATC1 | 1.15 | 0.013 | ↑ |
| JUN | 1.13 | 0.0005 | ↑ |

**
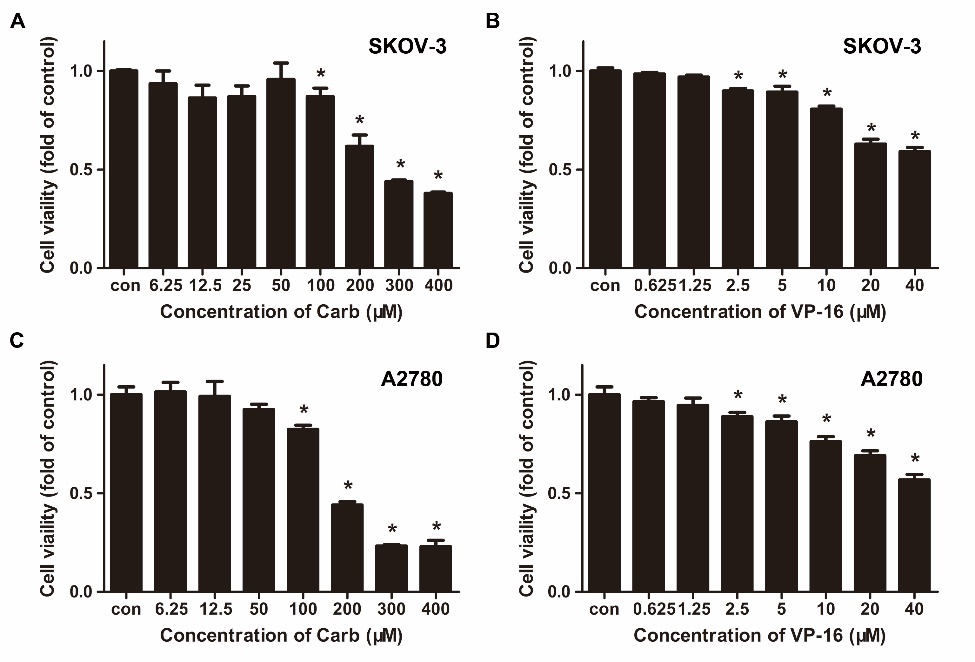
**

**Fig.S1** MTT assay of the carboplatin or VP-16 treated cells for 24 h. * p < 0.05 compared with control group.

**
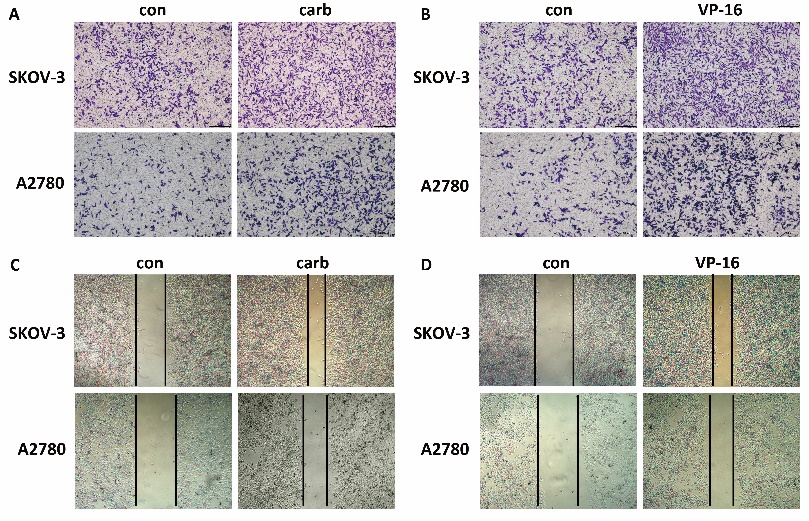
**

**Fig.S2 Chemotherapy exacerbated the migration of ovarian cancer cell lines.** SKOV-3 and A2780 cells were treated with carboplatin for 24 h and cultured for an extra of 72 h. Cell lines treated with VP-16 for 24 h were cultured for another 5-6 days. **(A-B)** Transwell migration assay were then conducted using the two cell lines respectively in the conditioned medium of the chemotherapy treated cells. The cells on the lower surface of the semipermeable membranes were fixed and stained by 0.1% crystal violet and photographed under the microscope. **(C-D)** Conditioned medium of the carboplatin or VP-16 treated cell lines were used in the wound healing assay of the SKOV-3 and A2780 cell lines. The figures are representatives of three experiments with replicates.


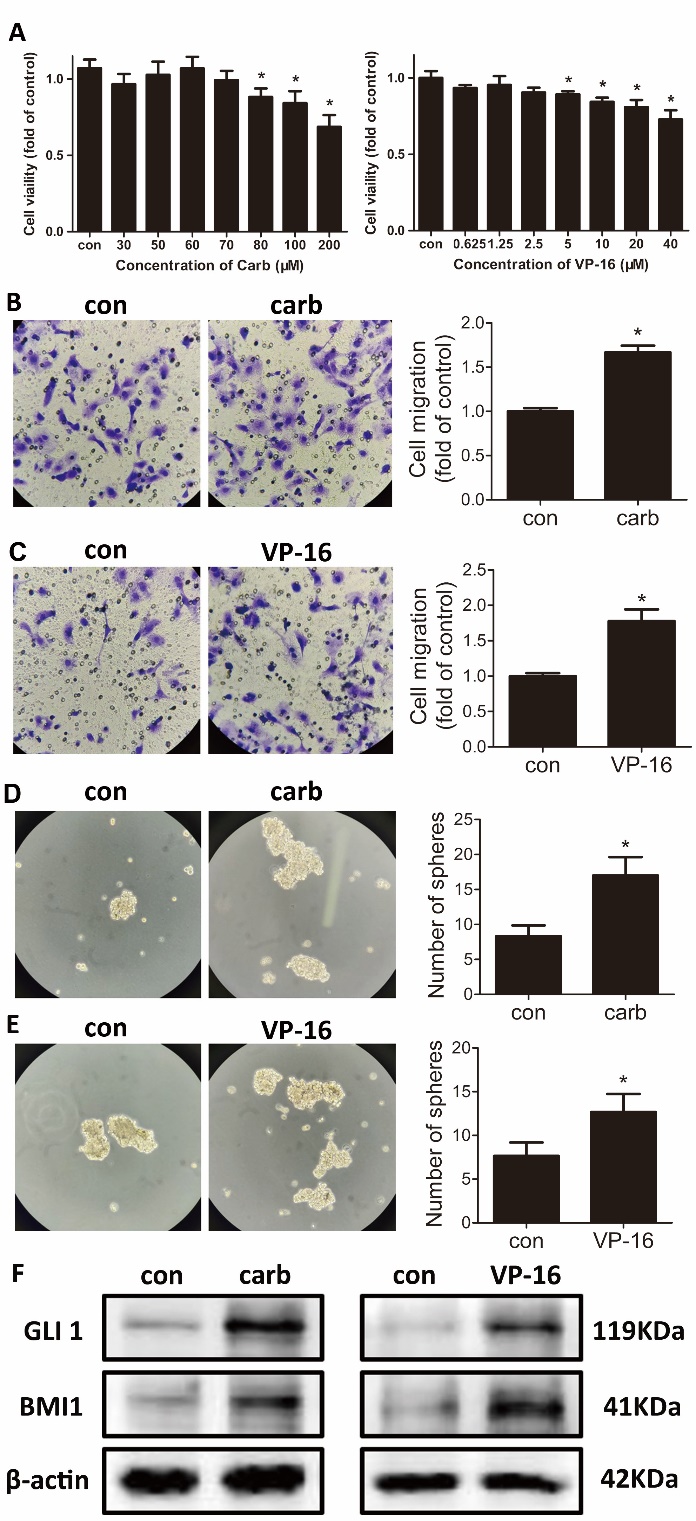


**Fig.S3 Chemotherapy exacerbated the migration, CSC-like characteristics and GLI1/BMI1 up-regulation in KURAMOCHI cell line.** KURAMOCHI cells were treated with carboplatin for 24 h and cultured for an extra of 72 h or treated with VP-16 for 24 h and cultured for another 4 days. **(A)** MTT assay of the carboplatin or VP-16 treated cells for 24 h. **(B-C)** Transwell migration assay were then conducted in the conditioned medium of the chemotherapy treated cells. The cells on the lower surface of the semipermeable membranes were fixed and stained by 0.1% crystal violet and photographed under the microscope. **(D-E)** The KURAMOCHI cell line co-cultured with the carboplatin or VP-16 treated cells were used in the sphere formation assay and cultured for 10 days. Colony diameters >50 μm were counted as a single positive colony. **(F)** After the 4 days’ co-culture, the receptor cells co-cultured with the carboplatin or VP-16 treated feeder cells were collected for western blot analysis. Results were expressed as the mean ± SD, * p < 0.05 compared with control group.


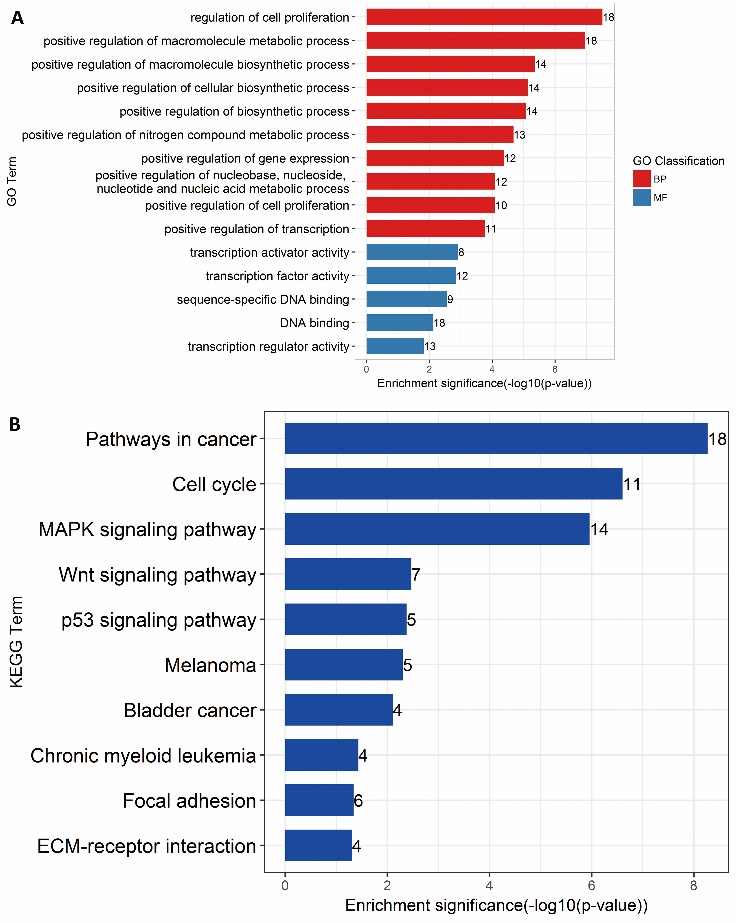


**Fig.S4** **KEGG and GO analysis of the differentially expressed genes (DEGs). (A)** KEGG analysis of the DEGs. **(B)** GO analysis of the DEGs.


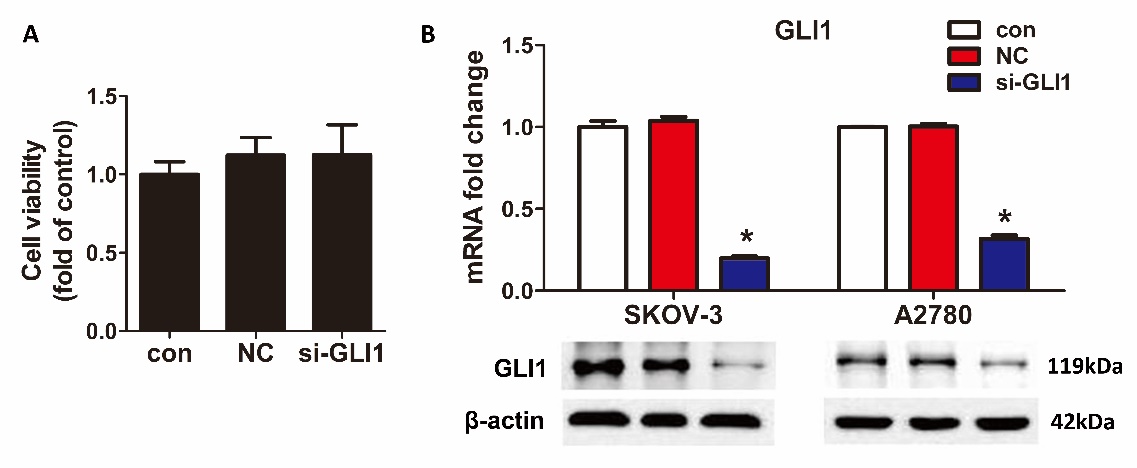


**Fig.S5 Transfection efficiency of GLI1 siRNA in SKOV-3 and A2780 cell lines. (A)** MTT assay of the GLI1 knockdown cells in 24 h. **(B)** After 48 hours’ incubation, the expression of GLI1 was detected by real-time quantitative PCR and western blot. * p < 0.05 compared with control group.


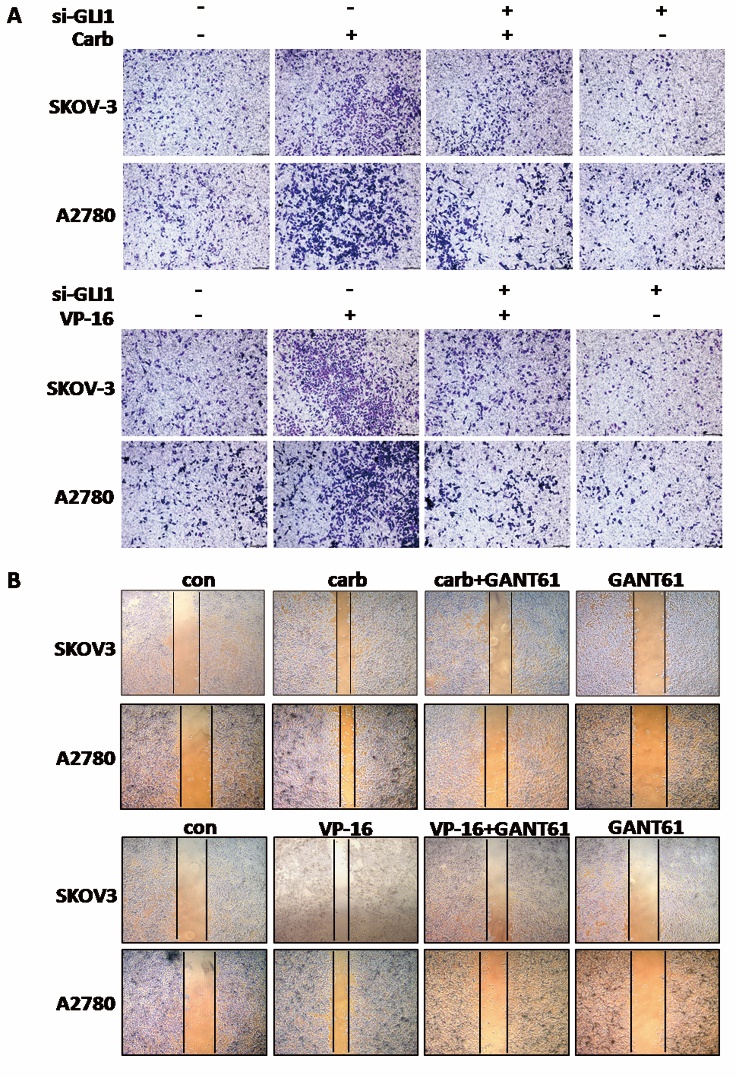


**Fig.S6 The images of the cells in the migration and wound healing assays photographed under the microscope.** **(A)** The cells on the lower surface of the membranes in the migration assay were fixed and then stained by 0.1% crystal violet. **(B)** The conditioned medium of the carboplatin or VP-16 treated cells was used in the wound healing assay. GANT61 was added simultaneously with the replacement of the conditioned culture medium. The figures are representatives of three experiments with replicates.


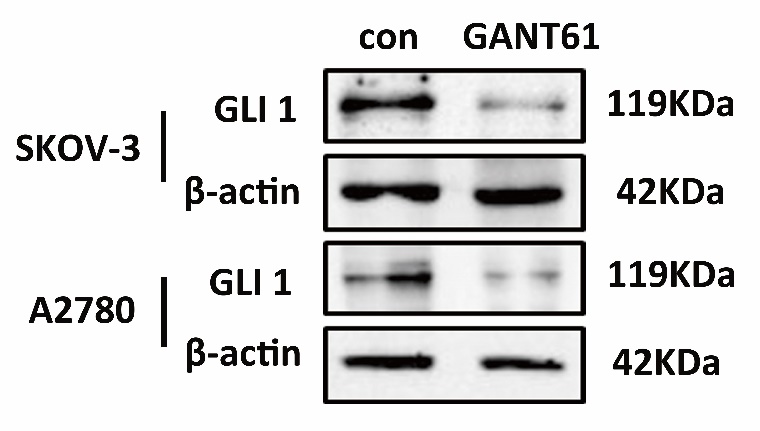


**Fig.S7 The expression of GLI1 48 h after GANT61 treatment.** SKOV-3 and A2780 cells were treated with GANT61 for 48 h and then collected for western blot analysis of GLI1 expression.
